# Guanine Nucleotides in the Meiotic Maturation of Starfish Oocytes: Regulation of the Actin Cytoskeleton and of Ca^2+^ Signaling

**DOI:** 10.1371/journal.pone.0006296

**Published:** 2009-07-20

**Authors:** Keiichiro Kyozuka, Jong T. Chun, Agostina Puppo, Gianni Gragnaniello, Ezio Garante, Luigia Santella

**Affiliations:** 1 Research Center for Marine Biology, Tohoku University, Asamushi, Japan; 2 Stazione Zoologica Anton Dohrn, Napoli, Italy; University of Hong Kong, Hong Kong

## Abstract

**Background:**

Starfish oocytes are arrested at the first prophase of meiosis until they are stimulated by 1-methyladenine (1-MA). The two most immediate responses to the maturation-inducing hormone are the quick release of intracellular Ca^2+^ and the accelerated changes of the actin cytoskeleton in the cortex. Compared with the later events of oocyte maturation such as germinal vesicle breakdown, the molecular mechanisms underlying the early events involving Ca^2+^ signaling and actin changes are poorly understood. Herein, we have studied the roles of G-proteins in the early stage of meiotic maturation.

**Methodology/Principal Findings:**

By microinjecting starfish oocytes with nonhydrolyzable nucleotides that stabilize either active (GTPγS) or inactive (GDPβS) forms of G-proteins, we have demonstrated that: *i*) GTPγS induces Ca^2+^ release that mimics the effect of 1-MA; *ii*) GDPβS completely blocks 1-MA-induced Ca^2+^; *iii*) GDPβS has little effect on the amplitude of the Ca^2+^ peak, but significantly expedites the initial Ca^2+^ waves induced by InsP_3_ photoactivation, *iv*) GDPβS induces unexpectedly striking modification of the cortical actin networks, suggesting a link between the cytoskeletal change and the modulation of the Ca^2+^ release kinetics; *v*) alteration of cortical actin networks with jasplakinolide, GDPβS, or actinase E, all led to significant changes of 1-MA-induced Ca^2+^ signaling.

**Conclusions/Significance:**

Taken together, these results indicate that G-proteins are implicated in the early events of meiotic maturation and support our previous proposal that the dynamic change of the actin cytoskeleton may play a regulatory role in modulating intracellular Ca^2+^ release.

## Introduction

The re-initiation of the meiotic cell cycle (maturation) of starfish oocytes can be resumed *in vitro* by adding the maturation hormone, 1-methyladenine (1-MA) [1]. Although the receptor of this hormone secreted by the follicle cells has not been identified, the effect of 1-MA appears to be transduced through guanine nucleotide-binding proteins (G-proteins). Support for this idea is provided by the inhibition of germinal vesicle breakdown (GVBD), which is the hallmark of meiotic maturation, by pertussis toxin [2]–[4]. It was later shown that the meiotic maturation of starfish oocytes is mediated by the βγ subunits of heterotrimeric G-proteins [5]–[7] through the activation of phosphoinositide 3-kinase and Akt kinase [8], [9].

The meiotic maturation of starfish oocytes starts with intracellular Ca^2+^ signaling. Within 1–2 min after addition of the hormone, free Ca^2+^ is quickly released from internal stores. This Ca^2+^ release takes place in the cortex of the vegetal hemisphere [10] and represents the first signaling event in the cell. Then, a dramatic structural reorganization of microvilli and the cortical actin network follows and is accompanied by a series of biochemical changes that characterize meiotic maturation [11], [12]. While studying the molecular mechanism underlying the intracellular Ca^2+^ release, we found that the Ca^2+^ release initiated much before any structural changes became evident in the endoplasmic reticulum (ER). This finding prompted a study for the roles of the actin cytoskeleton in the mobilization of Ca^2+^ in the cortical domain of the oocytes [10], [13], [14].

The detailed mechanism in which 1-MA induces Ca^2+^ release in starfish oocytes is not well known. Nonetheless, it could be inferred that the activation of heterotrimeric G-proteins by 1-MA would lead to the stimulation of PLCβ and thereby to the increased production of InsP_3_, the Ca^2+^-inducing second messenger [15]. However, our recent work indicated that the mechanism of Ca^2+^ release could be more complicated than this canonical model. We have found that the Ca^2+^-releasing mechanism induced by InsP_3_ was significantly influenced by changes of the actin cytoskeleton [16], [17]. Furthermore, treatment of starfish oocytes with the actin-depolymerizing agent latrunculin A (LAT-A) abolished the 1-MA-induced Ca^2+^ release [10]. The finding that inhibitors of the InsP_3_ pathway, e.g. heparin and U73122, caused alterations of the cortical actin cytoskeleton in starfish oocytes added weight to the suggestion that dynamic modulation of the actin cytoskeleton was also a crucial element in the regulation of intracellular Ca^2+^ signaling [10].

The finding that the 1-MA-induced meiotic maturation is mediated by G-proteins leads to the question whether the nucleotides acting on the G-proteins modulate the Ca^2+^ signaling in the way that is related to the changes of the actin cytoskeleton. Hence, in this contribution, we have investigated the effects of nonhydrolyzable analogues of GTP and GDP on the 1-MA-induced Ca^2+^ signaling patterns in starfish oocytes. As expected, we found that injection of oocytes with GTPγS mimicked the Ca^2+^-releasing effect of 1-MA. Conversely, the Ca^2+^ release was blocked by GDPβS injection. To our surprise, however, the preinjection of oocytes with GDPβS not only modulated the Ca^2+^ signaling pattern and kinetics, but also produced a significant enhancement of the actin network underneath the plasma membrane. This and other results in our study suggest that the dynamic change of the actin cytoskeleton is a crucial component of the mechanisms controlling intracellular Ca^2+^ signaling and cortical granule exocytosis.

## Materials and Methods

### 

#### Ethics statement. N/A

### Preparation of oocytes

The Japanese species of starfish (*A. pectinifera*) were captured in the Mutzu Bay (authorized by the Research Center for Marine Biology, Asamushi, Tohoku University, Japan) during the breeding season (September) and transported to the Stazione Zoologica in Naples, Italy. Animals were maintained in circulating cold seawater (16°C). The gonads containing numerous oocytes were dissected from the central dorsal area and transferred to cold filter-sterilized seawater. Fully grown immature oocytes were isolated as single cells by sieving through gauze several times. Nearly all the oocytes released from the gonads were arrested at the first prophase of meiosis, as judged by the presence of the germinal vesicle (nucleus). Free oocytes were isolated by repeated rinsing and low speed (<1,000 rpm) sedimentation in cold filtered seawater. For meiotic maturation, immature oocytes were stimulated with 1 µM of 1-MA for 1 h in filtered seawater.

### Microinjection, photoactivation of caged InsP_3_, and Ca^2+^ imaging

Microinjection of oocytes was performed with an air-pressure Transjector (Eppendorf). Typically, the amount of the injected material is estimated 1% of the oocyte volume. Hence, the final concentration of the injected material inside the oocyte is to be 100-fold lower than the concentration in the injection pipette. The fluorescent calcium dye (Oregon Green conjugated with 10 kDa dextran) was purchased from Molecular Probes, and was used in 5 mg/ml pipette concentration with the injection buffer (10 mM Hepes, pH 7.0, 0.1 M potassium aspartate). The same injection buffer was used for delivering caged InsP_3_ (Molecular Probes) by microinjection (50 µM, pipette concentration). To activate the caged InsP_3_, microinjected oocytes were irradiated with 330 nm UV light for 25 seconds with the use of the computer-controlled shutter system Lambda 10-2 (Sutter Instruments, Co., Novato, CA). Cytosolic Ca^2+^ changes were detected with a cooled CCD camera (MicroMax, Princeton Instruments, Inc., Trenton, NJ) mounted on a Zeiss Axiovert 200 microscope with a Plan-Neofluar 20x/0.50 objective. The quantified Ca^2+^ signal at a given time point was normalized to the baseline fluorescence (F_0_) following the formula F_rel_ = [F−F_0_]/F_0_, where F represents the average fluorescence level of the entire oocyte. Fluorescent Ca^2+^ images were analyzed with the MetaMorph Imaging System software (Universal Imaging Corporation, West Chester, PA, USA).

### F-actin staining and confocal microscopy

To visualize F-actin in living oocytes, the microinjection pipette was loaded with 50 µM Alexa Fluor 568-conjugated phalloidin in DMSO as previously described [10], [30]. Oocytes maintained in filtered seawater (FSW) were microinjected with the phalloidin probe and visualized with confocal microscopy after 10 min incubation. All steps were performed at room temperature. After staining, oocytes were transferred to an experimental chamber and were observed with an Olympus Fluoview 200 laser-scanning microscope with a 60× (1.20 NA) objective. Transmitted light and fluorescent confocal images were acquired from the equivalent cytoplasmic planes containing the GV. Images of F-actins stained with Alexa Fluor 568-conjugated phalloidin were recorded through a BP 510540 emission filter.

### Jasplakinolide, GDPβS, GTPγS, and denuded oocytes

GDPβS and GTPγS were purchased from Calbiochem, and jasplakinolide (JAS) from Molecular Probes. While JAS was dissolved in DMSO, GTPγS, GDPβS, and caged InsP_3_ were prepared in aqueous solution (injection buffer). The incubation conditions for jasplakinolide (JAS), GDPβS, and GTPγS were indicated in the figure legend. Denuded oocytes devoid of the vitelline coat were prepared by 30 minutes' incubation in 0.01% actinase E (750,000 tyrosine units/g; Kaken Chemical, Japan) at room temperature. Unless specified otherwise, the control cells refer to the oocytes that have been treated with the same vehicles for drug delivery.

## Results

### Intracellular Ca^2+^ release in starfish oocytes by 1-MA can be recapitulated by GTPγS microinjection

**Figure 1:**
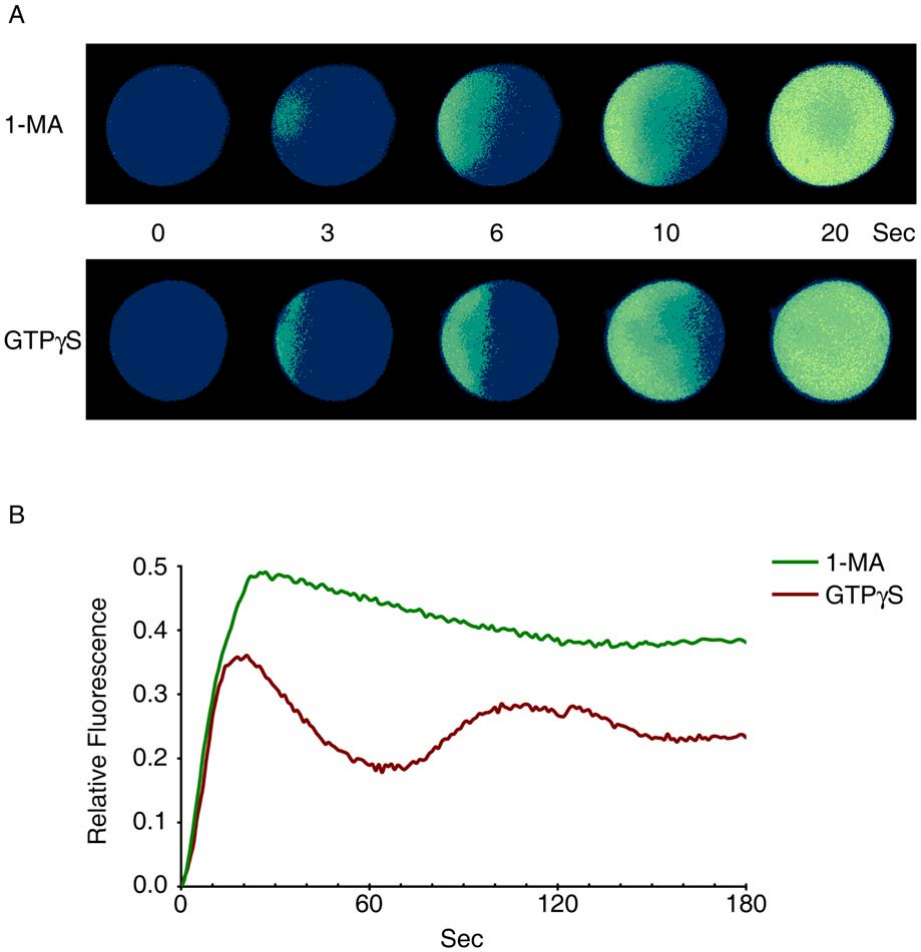
1-MA-induced Ca^2+^ signaling in starfish oocyte is mimicked by microinjection of GTPγS. *A. pectinifera* oocytes loaded with calcium dye were either incubated with 1-MA or microinjected with 50 mM of GTPγS. (A) Relative fluorescence pseudo-colored images of the Ca^2+^ indicator. To compare the kinetics of Ca^2+^ rise, the moment of the first detectable Ca^2+^ signal was set to t = 0 in both cases. (B) Quantification of intracellular Ca^2+^ levels induced by 1-MA (green curve) or GTPγS (brown curve).

The 1-MA-induced maturation of starfish oocyte is mediated by heterotrimeric G-proteins [2]–[7]. Microinjection of oocytes with GTPγS, a nonhydrolyzable analog of GTP, would thus be expected to simulate the effects of 1-MA on meiotic maturation and on Ca^2+^ release. To test if GTPγS can induce the same characteristic Ca^2+^ release seen in 1-MA-treated oocytes [10], the oocytes loaded with the Ca^2+^ dye were subjected to microinjection with GTPγS. As expected, GTPγS produced a very similar Ca^2+^ wave to that of the 1-MA-incubated oocytes. In both cases, the Ca^2+^ wave started from a single spot in the cortex of the vegetal hemisphere and propagated to the opposite side (Fig. 1A). However, the first Ca^2+^ response in GTPγS-injected oocytes appeared much later (127±15.4 sec, n = 8) than in 1-MA-treated oocytes (75.8±31.6 sec, n = 5) (*P*<0.05). This is likely due to the additional lag time required for the microinjected material to diffuse to the Ca^2+^ release sites in the oocyte cortex. Although the peak of the Ca^2+^ signal in GTPγS-injected oocytes displayed considerably lower amplitude (0.34±0.058 RFU, n = 8) than in 1-MA-incubated oocytes (0.48±0.077 RFU, n = 5) (*P*<0.01), the kinetics of the Ca^2+^ rise to the peak was strikingly similar in both cases (Fig. 1B). The time intervals from the first Ca^2+^ spot to the peak in GTPγS-injected and 1-MA-treated oocytes were 21.4±4.7 and 26.0±5.1 sec (*P*>0.05), respectively.

### Preinjection of GDPβS blocks 1-MA-induced Ca^2+^ signaling and GVBD in starfish oocytes

**Figure 2:**
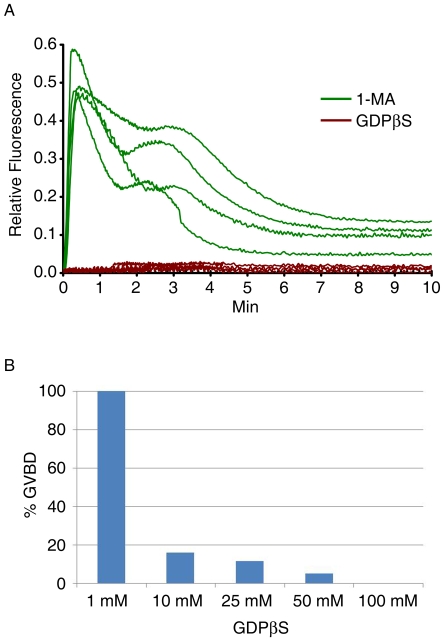
Preinjection of GDPβS blocks 1-MA-induced Ca^2+^ signaling and GVBD in starfish oocytes. *A. pectinifera* oocytes were microinjected with either GDPβS or the vehicle buffer and incubated for 15 min prior to the exposure to 1-MA. (A) Quantification of intracellular Ca^2+^ levels induced by 1-MA in the presence (brown curves, n = 4) or absence (green curves, n = 4) of GDPβS (100 mM, pipette concentration). (B) The effect of GDPβS on GVBD. Oocytes were injected with various amount of GDPβS and exposed to 1-MA for 1 h. The concentration of GDPβS in the histogram refers to the concentration in the microinjection pipette. The amount delivered into the oocyte was 1% of the cell volume. The frequency of oocytes that had undergone successful GVBD were calculated for each concentration of GDPβS (n = 84).

Starfish oocytes were then microinjected with GDPβS, a nonhydrolyzable analogue of GDP. As expected, preinjection of oocytes with GDPβS completely blocked 1-MA-induced Ca^2+^ response (Fig. 2A). Furthermore, microinjection of starfish oocytes with GDPβS inhibited the 1-MA-induced reinitiation of the cell cycle. The effect was dose-dependent, as judged by the percentages of oocytes undergoing germinal vesicle breakdown (GVBD) at various concentrations of GDPβS (Fig. 2B). Taken together, these results suggest that GDPβS can suppress the intracellular signaling system that transduces the effects of 1-MA at both early (Ca^2+^ signaling) and late (GVBD) stages of meiotic maturation.

### GDPβS microinjection alters the pattern of InsP_3_-induced Ca^2+^ release and the cortical actin cytoskeleton in immature oocytes

**Figure 3:**
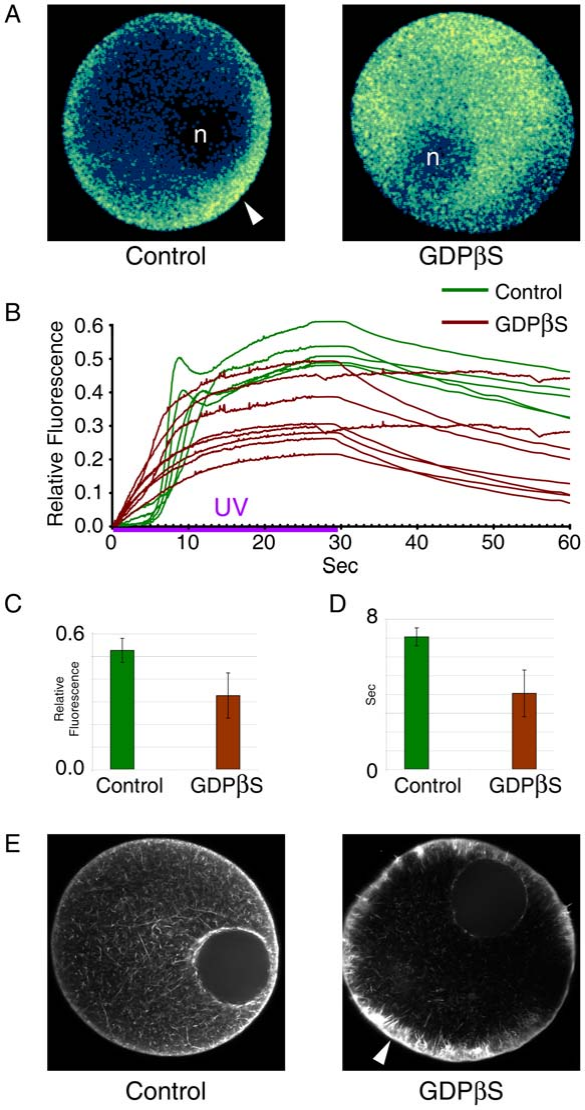
Effects of GDPβS on InsP_3_-dependent intracellular Ca^2+^ release and on the actin cytoskeleton in immature oocytes. *A. pectinifera* oocytes loaded with calcium dye and caged InsP_3_ were microinjected with GDPβS (100 mM, pipette concentration) or with the vehicle buffer. After 20 min incubation, the oocytes were illuminated with UV to uncage InsP_3_ and analyzed for intracellular Ca^2+^ release. To compare the kinetics of Ca^2+^ rise, the moment of the first detectable Ca^2+^ signal was set to t = 0 in both cases. (A) The relative fluorescence pseudo-colored images of the Ca^2+^ indicator at 7 sec. In control oocytes, InsP_3_-induced Ca^2+^ signals initiated from the cortex at the animal pole (arrow) near the nucleus (germinal vesicle, marked with n). In oocytes GDPβS-injected oocytes, this characteristic mode of Ca^2+^ wave initiation is lost. (B) Quantification of intracellular Ca^2+^ levels induced by uncaged InsP_3_ in the presence (brown curves, n = 8) or absence (green curves, n = 5) of GDPβS. The duration of photoactivation is marked with the violet bar labeled UV. (C) Comparison of the average Ca^2+^ peaks in the control (n = 5) and the GDPβS-injected (n = 8) oocytes (*P*<0.01). (D) Comparison of the kinetics of Ca^2+^ rises in the control (n = 5) and the GDPβS-injected (n = 8) oocytes. The time required for reaching 0.1 RFU was scored for each case, and the average and standard deviation of the values in the control and GDPβS-injected oocytes were presented in the histogram (*P*<0.001). (E) The state of the actin cytoskeleton in the control and GDPβS-injected oocytes. After 30 min incubation, actin filaments were visualized in live oocytes with Alexa Fluor 568-conjugated phalloidin (concentration in injection pipette, 50 µM). The arrowhead indicates enhancement of actin networks by GDPβS.

To test if GDP impairs the Ca^2+^ signaling mechanism of InsP_3_, starfish oocytes were microinjected with the same dose of GDPβS (100 mM, pipette concentration) that had totally eliminated the 1-MA-induced Ca^2+^ signaling. At variance with the effect on 1-MA-induced Ca^2+^ signals, GDPβS failed to block the Ca^2+^ wave that was generated by uncaged InsP_3_ (Fig. 3). Instead, the treatment conspicuously altered several aspects of the InsP_3_-induced Ca^2+^ release. Firstly, the hot spot of intense Ca^2+^ release at the cortex of the animal pole (Fig. 3A, arrowhead) was no longer visible in the GDPβS-injected oocytes. In the latter cells, Ca^2+^ signal evoked by InsP_3_ propagated without any preference for cell polarity (Fig. 3A, GDPβS). Furthermore, the initial Ca^2+^ release prominent in the cortex was also absent in these cells. Secondly, the kinetics of the Ca^2+^ rise by uncaged InsP_3_ is significantly changed in GDPβS-injected oocytes. While the amplitude of the Ca^2+^ peak was considerably reduced (0.33±0.10 RFU [Relative Fluorescence Unit], n = 8) in comparison with the control values (0.52±0.05 RFU, n = 5) (Fig. 3C), the latency period that characterizes the rise of the Ca^2+^ signals in control cells disappeared in GDPβS-injected cells (Fig. 3B). As a result, the time required for reaching 0.1 RFU in GDPβS-injected cells (4.03±1.24 sec) was much shorter than in the control cells (7.04±0.48 sec) (Fig. 3D). In parallel to these changes, we found that the treatment with GDPβS also strikingly altered the cortical actin networks in the same time-frame (Fig. 3E). While the phalloidin-stained F-actin fibers in the inner cytoplasm were slightly reduced in number, the F-actin networks in the subplasmalemmal region and the cortex were considerably enhanced in comparison with the control cells (Fig. 3E, arrowhead).

### GDPβS microinjection does not suppress InsP_3_-induced Ca^2+^ signals in postmeiotic eggs of starfish, but inhibits cortical granule exocytosis

**Figure 4:**
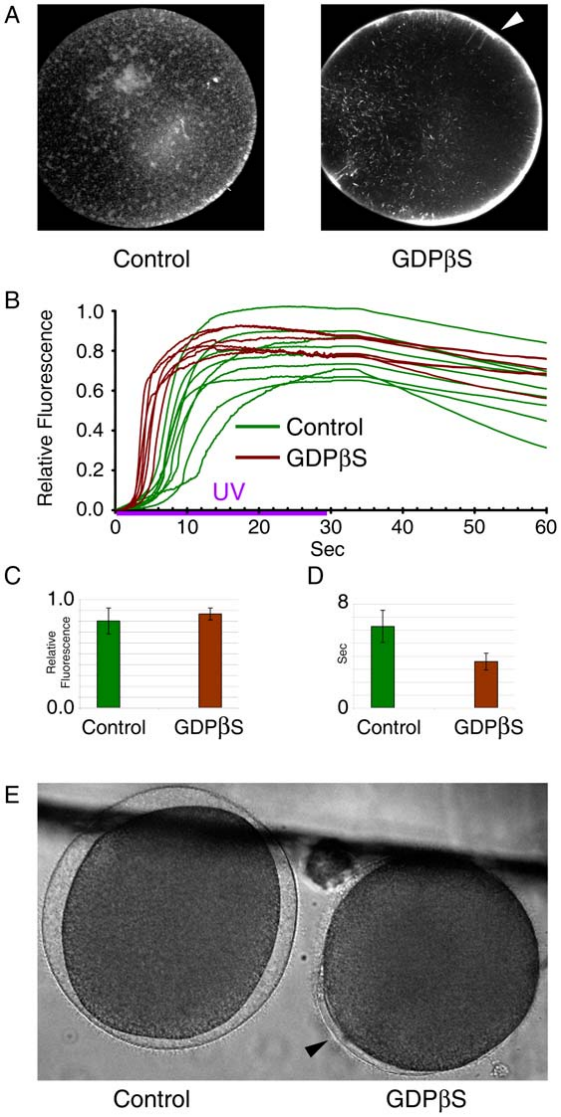
Effects of GDPβS on InsP_3_-dependent intracellular Ca^2+^ release and on the actin cytoskeleton in mature eggs. *A. pectinifera* oocytes were loaded with calcium dye and caged InsP_3_ and exposed to 1-MA for 1 h. The eggs displaying successful GVBD were microinjected with GDPβS (100 mM, pipette concentration) or with the vehicle buffer. After 20 min incubation, the oocytes were illuminated with UV to uncage InsP_3_ and analyzed for intracellular Ca^2+^ release. To compare the kinetics of Ca^2+^ rise, the moment of the first detectable Ca^2+^ release was set to t = 0 in both cases. (A) The state of the actin cytoskeleton in the control and GDPβS-injected eggs. After 30 min incubation, actin filaments were visualized with Alexa Fluor 568-conjugated phalloidin. The arrowhead indicates enhancement of cortical actin networks by GDPβS. (B) Quantification of intracellular Ca^2+^ levels induced by uncaged InsP_3_ in the presence (brown curves, n = 6) or absence (green curves, n = 9) of GDPβS. (C) Comparison of the average amplitude of the Ca^2+^ peaks in the control (n = 9) and the GDPβS-injected (n = 6) oocytes (*P*>0.1). (D) Comparison of the kinetics of Ca^2+^ rises in the control (n = 9) and the GDPβS-injected (n = 6) oocytes. The time required for reaching 0.1 RFU was scored for each case, and the average and standard deviation of the values in the control and GDPβS-injected eggs were presented in the histogram (*P*<0.001). (E) Elevation of vitelline layers in response to InsP_3_-induced intracellular Ca^2+^ release is largely blocked in the eggs pre-injected with GDPβS (n = 4). Partial elevation of the membrane is observed only in a limited area of the egg surface (arrowhead).

During meiotic maturation, starfish oocytes undergo a series of cytological changes that sensitize InsP_3_ receptors and rearrange the actin cytoskeleton [16], [18]. To examine the effects of GDP on these changes, we have microinjected postmeiotic eggs with the same amount of GDPβS that had blocked 1-MA-induced Ca^2+^ response. The results showed that the treatment enhanced the cortical actin networks underneath the plasma membrane (Fig. 4A). At variance with immature oocytes, GDPβS failed to affect the amplitude of the Ca^2+^ peaks generated in the postmeiotic eggs by the uncaging of InsP_3_ (Fig. 4B). The heights of the Ca^2+^ peaks in the control (0.80±0.12 RFU, n = 9) and the GDPβS-injected (0.86±0.055 RFU, n = 6) eggs were not significantly different (*P*>0.1) (Fig. 4C). However, microinjection of mature eggs with GDPβS had a significant effect on the kinetics of the Ca^2+^ rise. The time required for reaching 0.1 RFU in GDPβS-injected cells (3.54±0.65 sec, n = 6) was again much shorter than in the control (6.25±1.24 sec, n = 9, *P*<0.001) (Fig. 4D). Apparently, the ability to mobilize intracellular Ca^2+^ in response to InsP_3_ in postmeiotic eggs was not suppressed, but the Ca^2+^ response was rather quicker in the GDPβS-microinjected eggs. Nonetheless, the ability of these cells to support exocytosis of cortical granules was severely compromised, as judged by the failed elevation of the vitelline envelope (Fig. 4E).

### Both the 1-MA- and the GTPγS-induced Ca^2+^ signals are affected by the alteration of the actin cytoskeleton

**Figure 5:**
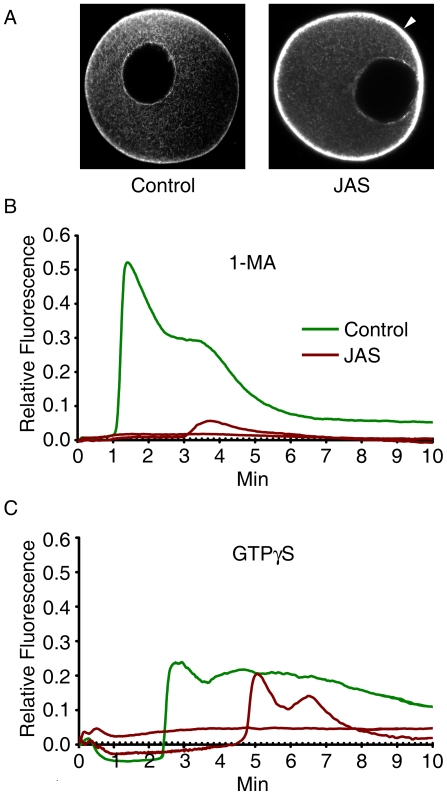
Effects of cortical actin networks on the Ca^2+^ waves generated by 1-MA and GTPγS. *A. pectinifera* oocytes loaded with calcium dye were incubated in the presence or absence of 12 µM JAS for 30 min and subjected to 1-MA or GTPγS treatments. (A) Alteration of cortical actin networks by JAS, as visualized by Alexa Fluor 568-conjugated phalloidin. Enhanced F-actin structures in the subplasmalemmal regions were marked with an arrowhead. (B) The Ca^2+^ response to 1-MA is nearly eliminated in JAS-treated oocytes. (C) The release of intracellular Ca^2+^ in response to GTPγS injection is either blocked or significantly delayed by JAS.

To test if the Ca^2+^ signals generated by 1-MA or GTPγS are influenced by the changes of the actin cytoskeleton, we have used jasplakinolide (JAS), a well-established agent provoking actin polymerization. JAS rearranges the actin cytoskeleton by inducing actin polymerization while inhibiting depolymerization of actin filaments *in vivo* [19], [10], [31]. Pre-incubation of starfish oocytes with JAS for 30 min heavily enhanced cortical actin networks (Fig. 5A, arrowhead) and almost completely blocked the 1-MA-induced Ca^2+^ signaling (Fig. 5B). Similarly, it also suppressed the Ca^2+^ response in GTPγS-injected oocytes (Fig. 5C). In some cases, however, JAS did not completely block but simply delayed the occurrence of the GTPγS-induced Ca^2+^ signals.

### The structure of the actin cytoskeleton and the 1-MA-induced Ca^2+^ signaling are altered in denuded oocytes without the vitelline coat

**Figure 6:**
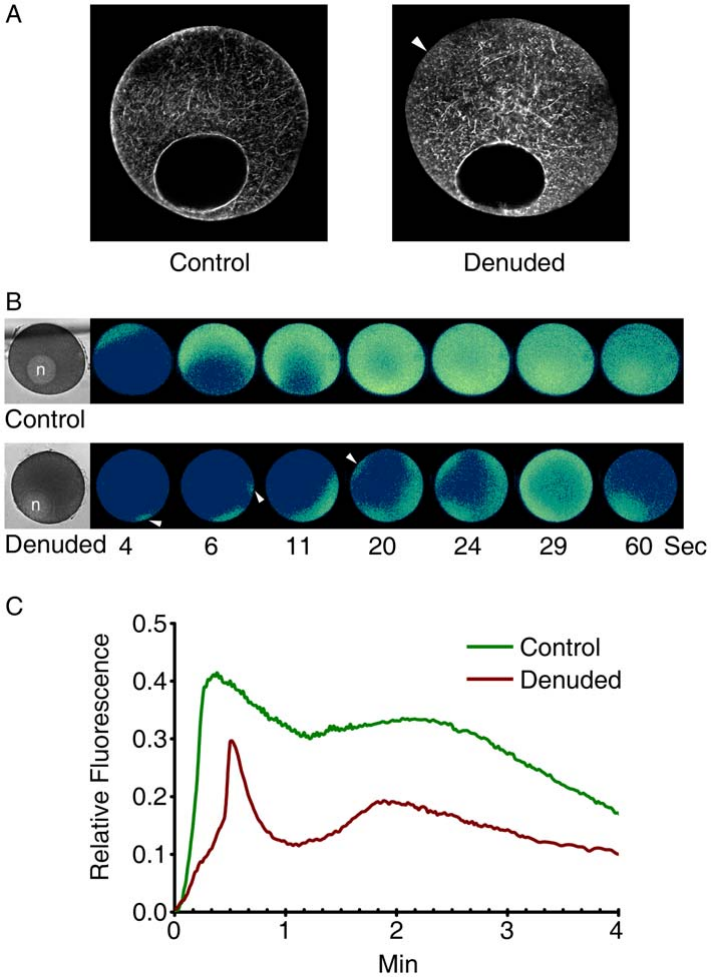
Alteration of the cortical actin network and propagation of Ca^2+^ signals in denuded oocytes. The vitelline coats of *A. pectinifera* oocytes were removed as described in Experimental Procedures. The denuded and intact (control) oocytes were then loaded with calcium dye and exposed to 1-MA. (A) Alteration of cortical actin networks in denuded oocytes, as visualized by Alexa Fluor 568-conjugated phalloidin. Abolishment of F-actin structures in the subplasmalemmal regions was marked with an arrowhead. (B) The relative fluorescence pseudo-colored images of the Ca^2+^ indicator after the addition of 1-MA. To compare the kinetics of Ca^2+^ rise, the moment of the first detectable Ca^2+^ signal was set to t = 0 in both cases. Ca^2+^ signals initiate at the vegetal hemisphere in control oocytes. In contrast, Ca^2+^ signals aberrantly arise at the animal pole near the nucleus (n) and from multiple spots (arrowheads) in the denuded oocytes. (C) Quantification of 1-MA-induced Ca^2+^ signals in the control (green) and denuded (brown) oocytes.

Starfish oocytes from which the vitelline coat was removed by the treatment with actinase E or trypsin still undergo meiotic maturation in response to 1-MA [20], [21]. Indeed, *A. pectinifera* oocytes denuded with actinase E treatment exhibited the same rate of GVBD as in intact oocytes [22]. This indicates that the removal of the vitelline coat still maintains functional 1-MA signaling pathways across the plasma membrane [23]. However, we have observed that the same treatment with actinase E results in subtle changes in the structure of the actin cytoskeleton. As shown in Fig. 6A, a 30 minute incubation of *A. pectinifera* oocytes in 0.01% actinase E selectively eliminated the cortical actin networks (arrowhead), with a slight ‘compensatory’ enhancement of F-actin bundles in the inner cytoplasm. These denuded oocytes still responded to 1-MA with a substantial Ca^2+^ release from the internal stores (Fig. 6B). However, the spatiotemporal kinetics of the Ca^2+^ rise was conspicuously different from that of the intact oocytes. First of all, the characteristic Ca^2+^ signaling patterns in the initial stage were quite deviant from the norm. Whereas the Ca^2+^ response in intact oocytes specifically started from a single spot in the cortex of the vegetal hemisphere, the addition of 1-MA to the denuded oocytes triggered Ca^2+^ signals near the animal pole and from multiple spots (Fig. 6B, arrowheads). Overall, the Ca^2+^ response in denuded oocytes were considerably irregular and lower in intensity, and the centripetal merge of the Ca^2+^ waves was less evident than in the control cells (Fig. 6B and C).

## Discussion

In this communication, we have first demonstrated that the 1-MA-induced Ca^2+^ mobilization inside starfish oocytes is, in large part, mimicked by GTPγS. The spatiotemporal pattern of the Ca^2+^ signals generated by the injection of GTPγS was virtually identical to that of 1-MA-evoked Ca^2+^ signals (Fig. 1). Furthermore, the analysis of the instantaneous increment of the Ca^2+^ signals indicated that the GTPγS-evoked Ca^2+^ release was largely restricted to the subplasmalemmal cortical region (not shown), as was the case with the 1-MA-induced Ca^2+^ wave propagation [10]. On the other hand, microinjection of oocytes with GDPβS has not only blocked propagation of 1-MA induced Ca^2+^ waves, but also inhibited progression of meiotic maturation (Fig. 2). Hence, our observations are in line with the notion that G-proteins are instrumental in the Ca^2+^ signaling and the progression of the meiotic maturation of starfish oocytes [5], [6], [10].

Although the βγ subunits of G-proteins have been implicated in Ca^2+^ signaling at fertilization of sea urchin eggs [24], the exact role of G-proteins in Ca^2+^ signaling at fertilization of deuterostomes remains controversial. Initial experiments in sea urchin eggs have shown that GTPγS induced cortical granule exocytosis, while GDPβS blocked sperm-induced cortical reaction [25], [26]. As Ca^2+^ chelators prevented GTPγS-mediated cortical granule exocytosis, Ca^2+^ was thought to be critical in the process [25]. By contrast, inhibition of G-proteins by injection of GDPβS failed to block the Ca^2+^ rise during fertilization, and even caused Ca^2+^ transients on its own [26]. Hence, the exact role of GDPβS inside the egg cell is still unclear.

Considering the broad targets of GTPγS and GDPβS inside the cell, it is conceivable that the effect of these nucleotides might have been mediated by downstream effectors other than heterotrimeric G-proteins. However, the specific inhibitors of Rho families of G-proteins such as *Clostridium difficile* toxin B [27] failed to inhibit 1-MA-induced Ca^2+^ signaling and the maturation process (not shown), arguing against the role of this type of monomeric G-proteins in meiotic maturation.

Our experimental data using GDPβS add significant new information to the molecular mechanism of Ca^2+^ signaling in maturing oocytes of starfish. Whereas the 1-MA-evoked Ca^2+^ release was completely blocked by GDPβS (Fig. 2A), the InsP_3_-dependent Ca^2+^ release mechanism was not (Fig. 3B). This discrepancy may reflect the difference in the ways how GDPβS influences the two Ca^2+^ release mechanisms. According to the prevailing view, GDPβS could reduce the 1-MA-induced synthesis of InsP_3_ by inhibiting PLCβ [15], [28] and thereby block the Ca^2+^ release, as was observed in Fig. 2A. On the other hand, since the Ca^2+^ release in response to photoactivation of exogenous InsP_3_ is independent of the PLC activity, GDPβS was not supposed affect the Ca^2+^ response by the uncaged InsP_3_. However, preincubation of immature oocytes with GDPβS substantially (36%) reduced the amplitude of the InsP_3_-evoked Ca^2+^ response (Fig. 3C). Hence, our results imply that GDPβS has an additional target (besides PLCβ) to influence Ca^2+^ signaling in starfish eggs.

What came out as the biggest surprise in our study was the striking effect of GDPβS on the cortical actin network. Examination of the living oocytes with Alexa Fluor 568-conjugated phalloidin showed that GDPβS had dramatically changed the structure of the cortical actin networks (Fig. 3E). The tight condensation of the cortical actin networks and the enhanced formation F-actin bundles, often perpendicular to the plasma membrane, are not the result of the alleged F-actin-stabilizing effect of phalloidin in living cells, as was demonstrated earlier with fixed cells [10].

Since it is well known that GTP can activate actin polymerization by stimulating the Rho/Rac/cdc42 family of G-proteins [29], our finding that not only GTP but also GDP can activate actin polymerization in the subplasmalemmal region of starfish oocytes may be somehow counterintuitive. However, the patterns of actin hyperpolymerization by GTPγS and GDPβS appear to be qualitatively different from each other. At variance with GDPβS, the treatment with GTPγS did not produce long actin fibers perpendicular to the egg plasma membrane. Furthermore, while the effect of GDPβS on the actin cytoskeleton persisted more than 30 min (Fig. 3E), the hyperpolymerized cortical actin in GTPγS-injected oocytes returned to the normal state after 15 min (Data S1). Although our results of GDP-based actin reorganization might imply a possible existence of a novel class of G-protein in the starfish eggs, which could promote actin polymerization in its GDP-bound conformation, such an idea is a matter of speculation at the current stage of our knowledge.

It has been known that other families of G-proteins, e.g. Rab and Arf, mediate vesicle trafficking [30]. Hence, our observation that GDPβS also significantly changes cortical actin networks complicated the interpretation of the GDPβS-induced blockade of the cortical granule exocytosis (Fig. 4E). While GDPβS may have silenced Rab-like G-proteins that may be at work in starfish eggs, it is also possible that the actin-related changes might have blocked cortical granule exocytosis in the same way as in JAS-treated eggs. Whatever the mechanism, it is clear is that the massive Ca^2+^ release in mature eggs is necessary, but not sufficient for cortical granule exocytosis [10], [31].

Recently, actin has been implicated in the regulation of intracellular Ca^2+^ signaling [10], [13], [14]. The conclusions on the InsP_3_-dependent intracellular Ca^2+^ signaling have been largely based on U73122 and heparin [32]. However, there have been many reports that cannot be explained by the InsP_3_-inhibitory effects of these two agents. In sea urchin eggs, U73122 (an inhibitor of PLC) and heparin (an antagonist of InsP_3_ receptors) blocked GTPγS-evoked Ca^2+^ transient [33], suggesting that GTPγS exerts its effect through InsP_3_-dependent pathways. In neuronal cell lines, however, InsP_3_ and GTPγS activated Ca^2+^ release by distinct mechanisms [34], implying that the mechanism by which GTPγS triggers Ca^2+^ release is still an open question. In support of the idea that tight regulation of the actin cytoskeleton should be considered in interpreting these data, we have found that both heparin and U73122 induce hyperpolymerization of cortical actin as well as blocking Ca^2+^ signaling [10]. Despite the reciprocal influence of the actin cytoskeleton and the local rise of free Ca^2+^, several lines of evidence have suggested that the status of actin polymerization can modulate the intracellular Ca^2+^-releasing mechanisms [14]. Firstly, we have demonstrated that the 1-MA-triggered Ca^2+^ release in starfish oocytes may be mediated by a novel mechanism involving F-actin and its associated proteins [10], [13], [14], [17], [35]. Secondly, sensitization of the Ca^2+^-releasing mechanism to InsP_3_ during meiotic maturation was strongly inhibited by latrunculin-A (LAT-A) [16]. We now show that the GDPβS-induced alteration of the actin cytoskeleton is correlated with the changes in both 1-MA- and InsP_3_-evoked Ca^2+^ signaling (Fig. 2, 3, and 4). The characteristic cortical Ca^2+^ response after InsP_3_ uncaging [16], [17] was altogether altered in GDPβS-injected oocytes, displaying faster Ca^2+^ response from broad regions (Fig. 3A and B). It is interesting that this conspicuous change in Ca^2+^ signaling mirrored the prominent hyperpolymerization of the cortical actin and the reduction of actin fibers in the inner cytoplasm (Fig. 3E). In support of a causal link between the quicker Ca^2+^ response and the reduced actin fibers in the cortex, LAT-A produced the similar effects [10]. Similarly, in the postmeiotic eggs, the structural changes of the cortical actin networks induced by GDPβS were accompanied by the functional changes of the InsP_3_-dependent Ca^2+^-releasing mechanism, as judged by the changes of the kinetics in the Ca^2+^ rise (Fig. 4). In addition, the subtle changes in the cortical actin cytoskeleton of the denuded oocytes were also associated with the changes of Ca^2+^ signaling, further advocating this view on the role of actin in Ca^2+^ signaling (Fig. 6). As the structure of the cortical actin cytoskeleton was altered prior to examining the patterns of Ca^2+^ signaling, the temporal relationship between the actin and Ca^2+^ changes clearly supports the idea of actin-based modulation of Ca^2+^ signaling in these cases.

Given that GDPβS can interact with many downstream partners, the alteration of the actin cytoskeleton may not be the sole factor responsible for the changes in the Ca^2+^ signaling pattern. However, the finding that the actin-polymerizing agent JAS inhibited the 1-MA-induced Ca^2+^ response and the GTPγS-evoked Ca^2+^ release (Fig. 5) can only be explained by the actin changes [10]. The example of the actin-based modulation of intracellular Ca^2+^ signaling was also provided in other cells such as hippocampal neurons, where the ER-based Ca^2+^ release was significantly potentiated by jasplakinolide and attenuated by cytochalasin D [36]. While the hippocampal neurons treated with jasplakinolide apparently expedited the Ca^2+^ response [36], we have observed jasplakinolide produce similar effects in fertilized eggs of starfish (Data S2).

What is not clear is how GDPβS affects several aspects of Ca^2+^ signaling in response to InsP_3_. As InsP_3_ receptor is anchored to the actin cytoskeleton [37], it is conceivable that the reduced amplitude of Ca^2+^ peak might be attributed to the modulation of the efficacy of InsP_3_ receptors by cytoskeletal changes. On the other hand, it is more difficult to understand what has expedited the Ca^2+^ response in the GDPβS-treated eggs (Fig. 3 and 4). In theory, the changing actin cytoskeleton might have altered the kinetics of Ca^2+^ release by modifying its ‘allosteric’ influence on InsP_3_-receptors, or by redistributing the receptors attached to the ER membrane [38], [36], [14]. Alternatively, disparate reorganization of the actin pools in the cortex might have created a certain microenvironment that facilitates diffusion of InsP_3_ and Ca^2+^.

GDPβS may also exert its effect on Ca^2+^ signaling in other unknown pathways. A recent study from pancreatic acinar cells has suggested that Gβγ may either prevent InsP_3_ from binding to InsP_3_ receptor or gate its Ca^2+^ channeling moiety by a novel mechanism involving direct physical interaction [39]. If a similar signaling pathway were at work in starfish oocytes, it would affect the InsP_3_-evoked Ca^2+^ rise. However, the existence of such a collateral pathway of Gβγ in the physiological context of starfish eggs should be further investigated. Suggesting a more direct functional link between the actin cytoskeleton and G-proteins, Gβγ subunits were found to be physically associated with actin filaments in several cell lines [40]. Future attempts to determine the physiological relevance of such a liaison between heterotrimeric G-proteins and actin are likely to provide insights into the new roles of these two ubiquitous proteins in cell.

## Supporting Information

Data S1 Hyperpolymerization of cortical actin by GTPγS. Immature oocytes of *A. pectinifera* were microinjected with salt-matching buffer (control) or 100 mM GTPγS. The actin changes were monitored by subsequent microinjection of Alexa Fluor 568-phalloidin respectively 3 and 15 min after the GTPγS injection.
(0.27 MB PDF)

Data S2 Jasplakinolide-treated eggs display quicker release of Ca^2+^ in response to fertilizing sperm. The postmeiotic eggs of *A. pectinifera* loaded with Ca^2+^ dyes were fertilized after 15 min incubation in the presence (violet curves) or absence (green curves) of 6 µM of jasplakinolide. The Ca^2+^ response to the fertilizing sperm is substantially faster in jasplakinolide-treated eggs.
(0.04 MB PDF)
