# Supplementary figures and images for: Guanine Nucleotides in the Meiotic Maturation of Starfish Oocytes: Regulation of the Actin Cytoskeleton and of Ca2+ Signaling
Source: PLoS One. 2009 Jul 20;4(7):e6296. doi: 10.1371/journal.pone.0006296 (PMC2706993; doi:10.1371/journal.pone.0006296)

**Control**

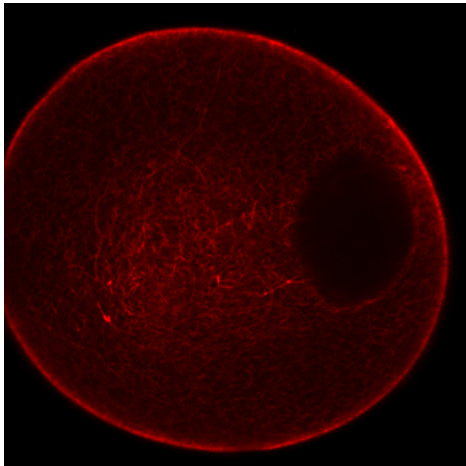

**GTP $\gamma$ S (3 min)**

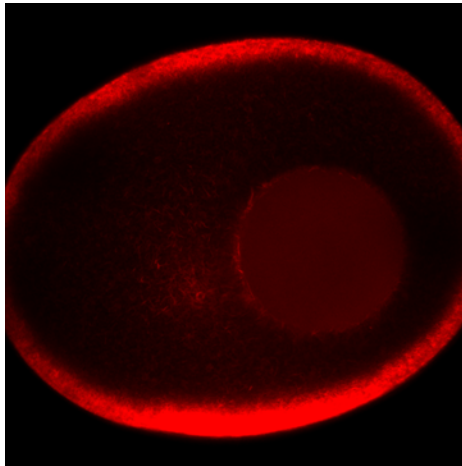

**GTP $\gamma$ S, (15 min)**

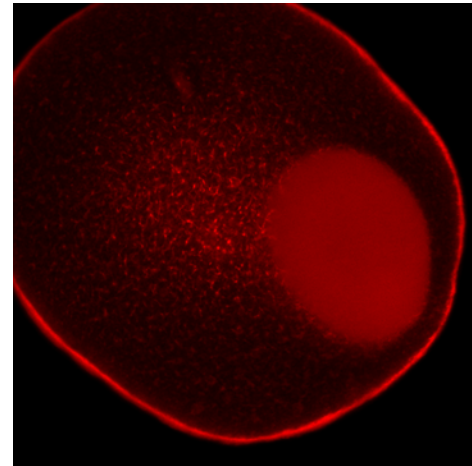

**(Supplementary data 1)**

Supplement: Data S1 — Hyperpolymerization of cortical actin by GTPγS. Immature oocytes of A. pectinifera were microinjected with salt-matching buffer (control) or 100 mM GTPγS. The actin changes were monitored by subsequent microinjection of Alexa Fluor 568-phalloidin respectively 3 and 15 min after the GTPγS injection. (0.27 MB PDF) [file pone.0006296.s001.pdf]

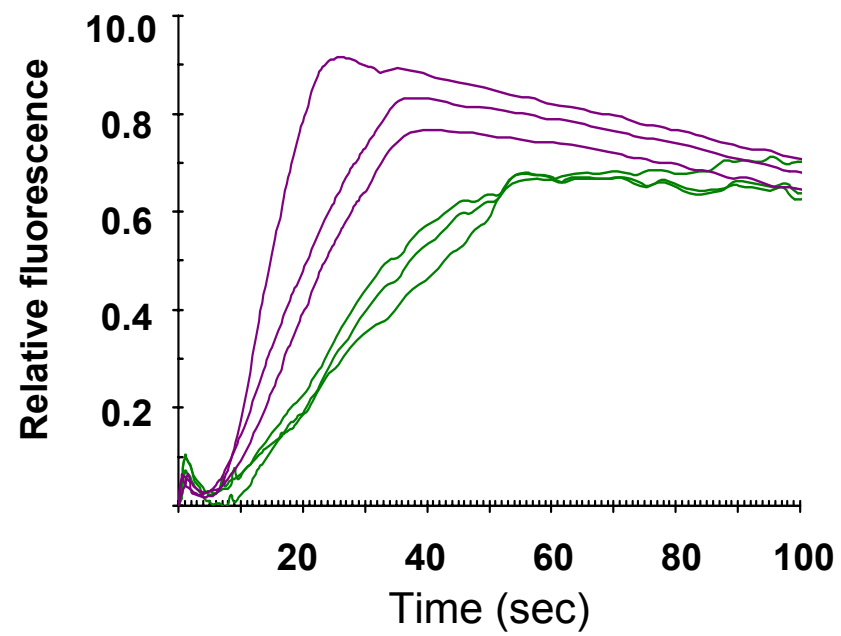

**(Supplementary data 2)**

Supplement: Data S2 — Jasplakinolide-treated eggs display quicker release of Ca2+ in response to fertilizing sperm. The postmeiotic eggs of A. pectinifera loaded with Ca2+ dyes were fertilized after 15 min incubation in the presence (violet curves) or absence (green curves) of 6 µM of jasplakinolide. The Ca2+ response to the fertilizing sperm is substantially faster in jasplakinolide-treated eggs. (0.04 MB PDF) [file pone.0006296.s002.pdf]
